# Safety, immunogenicity and efficacy of an mRNA-based COVID-19 vaccine, GLB-COV2-043, in preclinical animal models

**DOI:** 10.1038/s41598-023-46233-6

**Published:** 2023-12-01

**Authors:** Felipe Lelis, Laura A. Byk, Sergei Pustylnikov, Vivian Nguyen, Brandon Nguyen, Malorie Nitz, Prutha Tarte, Kunal Tungare, Jilong Li, Saikat Manna, Sampa Maiti, Dhwani H. Mehta, Narendran Sekar, Diana M. Posadas, Himanshu Dhamankar, Jeffrey A. Hughes, Lorenzo Aulisa, Amin Khan, Mariane B. Melo, Antu K. Dey

**Affiliations:** 1 GreenLight Biosciences Inc., 29 Hartwell Avenue, Lexington, MA 02421 USA; 2 Present Address: Pharmaron, Woburn, MA USA; 3 Present Address: Sanofi, Waltham, MA USA; 4 Present Address: Sanofi, Cambridge, MA USA; 5 Present Address: Invaio, Cambridge, MA USA; 6 Present Address: CRISPR Therapeutics, Boston, MA USA

**Keywords:** Infectious diseases, Vaccines

## Abstract

Several COVID-19 vaccines, some more efficacious than others, are now available and deployed, including multiple mRNA- and viral vector-based vaccines. With the focus on creating cost-effective solutions that can reach the low- and medium- income world, GreenLight Biosciences has developed an mRNA vaccine candidate, GLB-COV2-043, encoding for the full-length SARS-CoV-2 Wuhan wild-type spike protein. In pre-clinical studies in mice, GLB-COV2-043 induced robust antigen-specific binding and virus-neutralizing antibody responses targeting homologous and heterologous SARS-CoV-2 variants and a T_H_1-biased immune response. Boosting mice with monovalent or bivalent mRNA-LNPs provided rapid recall and long-lasting neutralizing antibody titers, an increase in antibody avidity and breadth that was held over time and generation of antigen-specific memory B- and T- cells. In hamsters, vaccination with GLB-COV2-043 led to lower viral loads, reduced incidence of SARS-CoV-2-related microscopic findings in lungs, and protection against weight loss after heterologous challenge with Omicron BA.1 live virus. Altogether, these data indicate that GLB-COV2-043 mRNA-LNP vaccine candidate elicits robust protective humoral and cellular immune responses and establishes our mRNA-LNP platform for subsequent clinical evaluations.

## Introduction

The coronavirus disease 2019 (COVID-19) pandemic has significantly impacted global health and economies^1^. This highlighted the urgent need for accelerated development and deployment of effective vaccines to prevent infection and the spread of the severe acute respiratory syndrome coronavirus 2 (SARS-CoV-2) identified in 2019 in Wuhan, China^2,3^. Despite the rapid development and authorization under emergency use of several platform-based COVID-19 vaccines that proved to be safe and effective against disease severity, hospitalizations^4–8^, the inequitable distribution of these vaccines contributed to higher vaccine coverage in developed countries in comparison to compared to low- and medium- incoming countries^9–12^. The development and administration of safe and effective booster vaccines remain key to augmenting the protective response against the emerging variants of the virus and their equitable deployment is essential to mitigating the pandemic's toll on global health and economies^13–15^.

In response to the COVID-19 pandemic, the first-generation mRNA vaccines, encoding for a prefusion stabilized version of the spike (S) protein of SARS-CoV-2 wild-type (Wuhan-Hu-1) strain, were developed by Moderna and Pfizer-BioNTech^5,16,17^. These vaccines were safe and highly effective in preventing severe COVID-19 disease, hospitalization, and death in clinical trials, and therefore authorized for emergency use in humans^5,6^. In this process, the mRNA technology was able to demonstrate the fastest vaccine development ever seen, suitable for emerging infectious diseases that require a rapid response in situations of local outbreaks or global pandemics^18–21^. Furthermore, the mRNA vaccine platform is now being used in vaccine development against several other infectious diseases, such as Shingles, RSV, HIV, and Malaria, particularly because of its cost and time-effective advantages compared to traditional vaccine platforms^22–24^.

Previously, our team showed preliminary data that GreenLight Biosciences’ COVID-19 mRNA vaccine candidate, GLB-COV2-043, is immunogenic and conferred protection upon challenge by the Wuhan strain of SARS-CoV-2 in preclinical studies^25^. In this study, we reinforce those findings and evaluate the potential of our vaccine candidate as a booster dose (a third dose), in mice, and measure its long-term memory responses and durability of binding and neutralizing antibodies against homologous strain and several heterologous variants of SARS-CoV-2. We also studied the effectiveness of the booster dose in protection against SARS-CoV-2 Omicron BA.1 challenge in hamsters. Our data suggests that GLB-COV2-043 elicits short and long-term potent humoral and cellular immune responses in C57BL/6 mice. Besides, GLB-COV2-043 also proved to be efficacious in protecting Golden Syrian hamsters in a challenge model against Omicron BA.1 virus. Finally, we performed a cGLP Toxicology study in Sprague Dawley Rats and found that GLB-COV2-043 was well-tolerated and effects attributed were consistent with the immunological and inflammatory changes associated with the intramuscular administration of an immunogenic mRNA vaccine. These data, taken in context of our modified mRNA and lipid-nanoparticle (LNP) delivery technology, establishes our mRNA-LNP platform as a viable platform technology for future vaccine development against infectious disease targets, including emerging and re-emerging pathogens.

## Results

### In vitro characterization of GLB-COV2-043 mRNA

**Figure 1:**
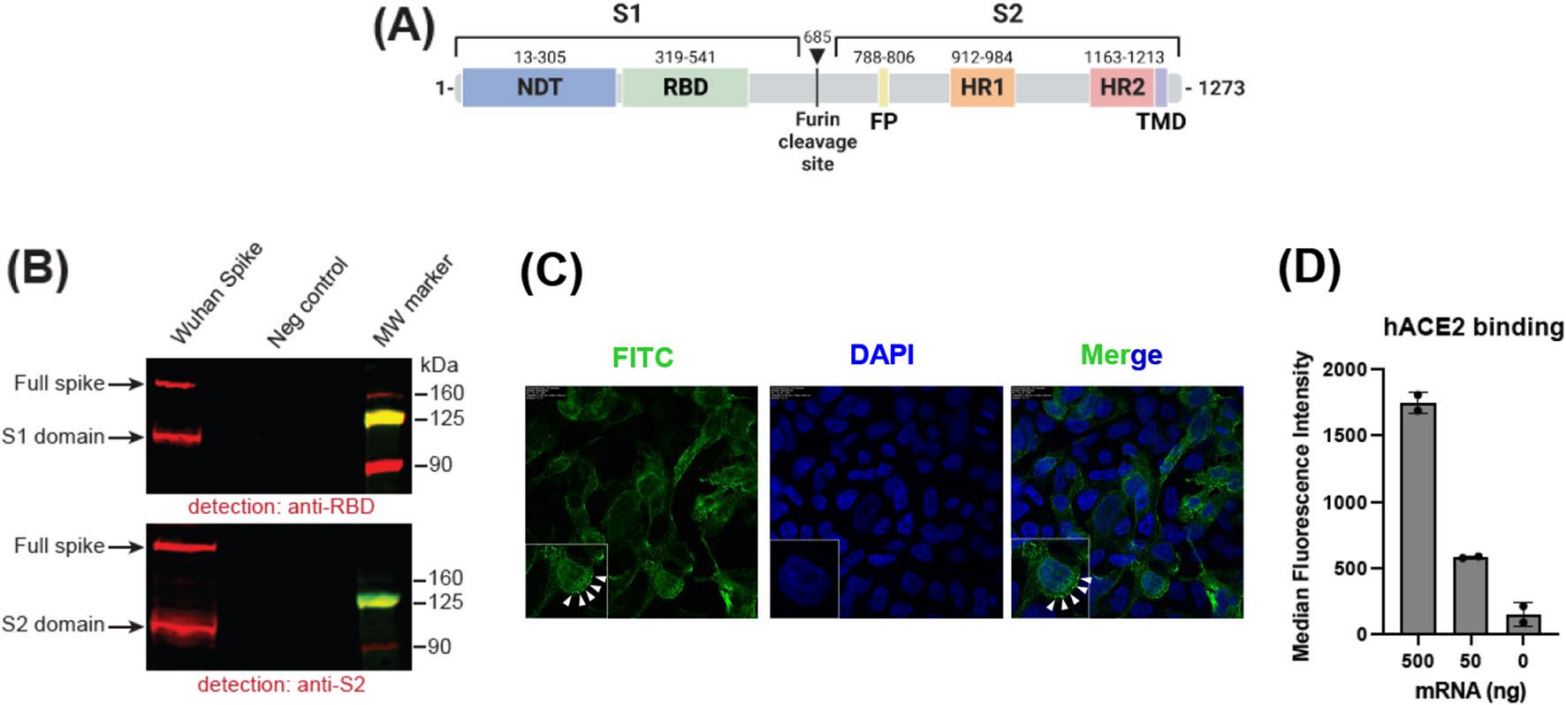
Immunogen design and in vitro characterization. (**A**) Schematic representation of the domain structure of wild-type full-length SARS-CoV-2 Wuhan spike protein. The residues that constitute the S1 and S2 domains are indicated. NTD, N-terminal domain; RBD, receptor binding domain; FP, fusion peptide; HR1/HR2, heptad-repeats; TMD, transmembrane domain. The arrow denotes furin cleavage site. (**B**). Detection of the spike protein in cells lysates. HEK293FT cells were transfected with mRNA expressing SARS-CoV-2 wild-type full-length spike and evaluated by western blot 24 h post-transfection using anti-RBD (upper panel) or anti-S2 domain antibodies (lower panel). Mock-transfected (PBS) cells served as a negative control. Full spike, full length spike; MW, molecular weight. (**C**) HEK293FT cells were transfected with mRNA expressing SARS-CoV-2 wild-type spike and evaluated by confocal microscopy 24 h post-transfection. The expressed spike(S) protein were detected with anti-S rabbit IgG, which in turn were detected using anti-rabbit-IgG-FITC (green). The nuclei were stained with DAPI (blue). The FITC-DAPI merged images are shown to highlight the expression of the Spike (S) protein on cell surface and highlighted by arrows (in both FITC and Merged panels) (**D**) Binding of recombinant human ACE2 (hACE2) to cell-surface expressed spike. HEK293FT cells were transfected with the indicated amounts of mRNA encoding for spike and binding to recombinant hACE2 protein was evaluated by flow cytometry 24 h post-transfection. Data are presented as median ± standard deviation. Mock-transfected (PBS buffer) cells served as a negative control.

We developed GLB-COV2-043, a pseudouridine(Ψ)-modified mRNA encoding for full-length spike (S) protein of the Wuhan SARS-CoV-2 (Wuhan-Hu-1) virus. This mRNA construct is composed of: (i) a transcription initiation sequence (ITS), developed by GreenLight Biosciences, to increase production yields of the RNA, (ii) human hemoglobin beta (HBG) 5’UTR and 3’UTR sequences and (iv) a 100 bp long polyA tail sequence (Fig. 1A). To demonstrate full-length spike protein expression, in vitro transcribed mRNA was transfected into HEK293FT cells, and protein expression was subsequently analyzed by Western blot. In this in vitro system we successfully detected expression of full-length spike, and cleaved S1 and S2 domains (Fig. 1B and Fig. S1). To confirm surface expression of the full-length spike protein, we used confocal microscopy. HEK293FT cells were transfected with mRNA expressing SARS-CoV-2 wild-type full-length spike and evaluated by confocal microscopy 24 h post-transfection, without permeabilization. The expressed spike(S) protein was detected as punctuated dots at the cell surface (Fig. 1C). Similar data, demonstrating expression of full-length spike, was also observed using flow cytometry (not shown). In addition, to assess functionality of the expressed spike protein, we transfected HEK293FT cells with varying amounts (50 ng and 500 ng) of GLB-COV2-043 mRNA and evaluated its ability to bind to its natural cell entry receptor, human angiotensin-converting enzyme 2 (hACE2), by flow cytometry. As observed in Fig. 1D, membrane-bound full-length spike protein (expressed from GLB-COV2-043 mRNA) on HEK293FT effectively bound to hACE2.

### Primary immunization with GLB-COV2-043 induces strong binding and neutralizing antibodies against SARS-CoV-2 in mice

**Figure 2:**
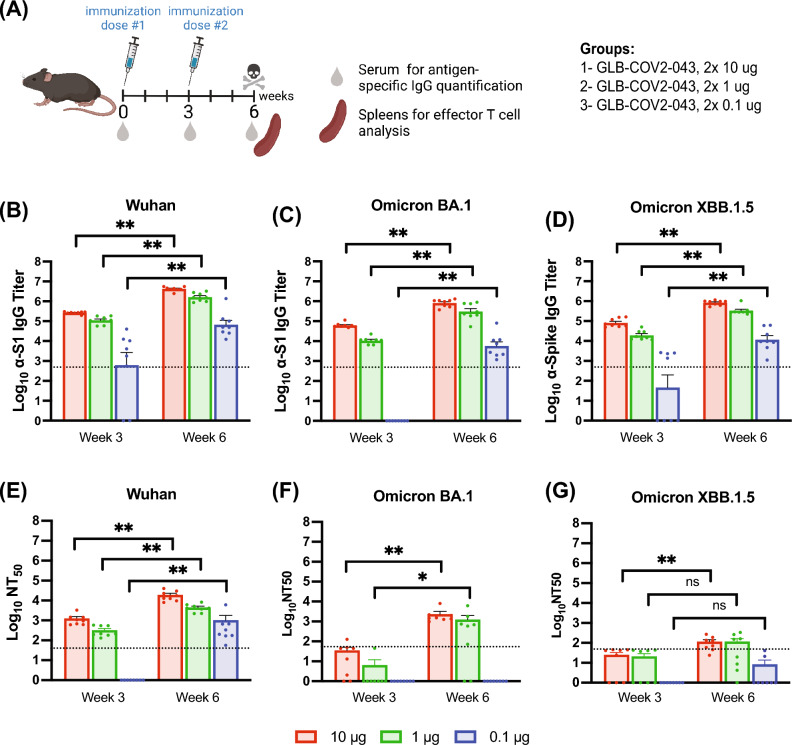
Primary immunization with GLB-COV2-043 mRNA-LNP vaccine induces strong binding and neutralizing antibodies against SARS-CoV-2 Wuhan and Omicron subvariants in a dose-dependent manner. C57BL/6 mice were vaccinated intramuscularly with two doses of 10 µg or 1 µg or 0.1 µg mg, three weeks apart, of GLB-COV2-043 mRNA-LNP. (**A**) Scheme of immunization and sample collection schedule. (B-D) End-point titers of antigen-specific serum IgG antibodies detected in sera collected from mice at indicated timepoints and evaluated by ELISA using soluble S1 recombinant spike proteins from Wuhan (**B**), Omicron BA.1 (**C**) or Omicron XBB.1.5 (**D**) SARS-CoV-2 variants. (**E**–**G**) Serum anti-SARS-CoV-2 neutralization titers were evaluated by pseudovirus neutralization tests using SARS-CoV-2 homologous (**E** Wuhan) and heterologous (**F** Omicron BA.1 and **G** Omicron XBB.1.5) pseudotyped viruses. The Mean ± SEM is shown, and dotted lines indicate LLOQ (Lower Limit of Quantitation). All dose levels were compared by Kruskal–Wallis Analysis of variance (ANOVA) followed by Dun’s test multiple comparisons. Two-sided Wilcoxon signed rank test. *p < 0.05 **p < 0.01 ns: not significant.

Levels of circulating binding and neutralizing antibodies to SARS-CoV-2 have been associated with protection from severe outcomes of COVID-19 and hospitalization in vaccine recipients and are known to predict immunogenicity, safety, and vaccine efficacy^26–28^. Measuring binding and neutralizing antibodies induced by GLB-COV2-043 in our preclinical studies was a key parameter to investigate its immunogenicity. Thus, an initial study was designed to assess the humoral immune responses induced by GLB-COV2-043 through a 2-dose prime vaccination scheme in mice. Briefly, C57BL/6 female mice were intramuscularly immunized on days 0 and 21 with the GLB-COV2-043 mRNA-LNP vaccine (Fig. 2A). Mice were separated into three groups that received escalating doses of the mRNA-LNP vaccine (0.1 µg, 1 µg, and 10 µg). A control group received saline. Sera, either from week 3 or week 6 post immunization, demonstrated robust homologous and heterologous serum binding IgG to Wuhan and Omicron BA.1 S1 subunit, and to Omicron XBB.1.5 S full-length spike proteins, in a dose-dependent fashion, as measured by ELISA (Fig. 2B–D). In the group that received the lowest dose of 0.1 µg, nearly all mice seroconverted against the Wuhan S1 protein, with a mean value above the lower limit of quantification (LLOQ), shown by the dotted line in Fig. 2B. The second immunization resulted in a 1.5-fold increase, on average, in anti-S1 serum IgG titer. A similar increase was also observed in homologous anti-S1 serum binding IgG levels from animal groups that received 1 µg or 10 µg of GLB-COV2-043 mRNA-LNP (Fig. 2B). Although no mice seroconverted against Omicron BA.1 S1 after a single low dose of 0.1 µg of GLB-COV2-043 mRNA-LNP vaccine, all immunized animals presented high anti-BA.1 S1 serum IgG titers post second dose (Fig. 2C). To assess anti-S1 antibody binding breadth induced by GLB-COV2-043 mRNA-LNP immunization, we also measured serum antibody binding titers against a most recent circulating variant of SARS-CoV-2, Omicron XBB.1.5. After initial immunization, half of the animals dosed with 0.1 µg of the mRNA-LNP vaccine seroconverted, while all animals dosed with 1 or 10 µg of vaccine developed high serum titers of anti-XBB.1.5 spike IgG. (Fig. 2D). At week 6, all mice in all groups showed high levels of anti-XBB.1.5 spike binding IgG. A saline group was used as a negative control, and at all time points, the binding titers against all antigens tested were undetectable (data not shown).

To evaluate the ability of serum anti-SARS-CoV-2 antibodies induced by our mRNA-LNP vaccine to block viral entry into host cells, we performed pseudovirus neutralization assays using commercially available pseudotyped viral particles with a Murine leukemia virus (MLV) backbone expressing the Wuhan, Omicron BA.1 or XBB.1.5 SARS-CoV-2 spike proteins and containing a luciferase reporter gene. HEK293T cells expressing the hACE2 receptor were used for pseudovirus infectivity and antibody-neutralizing activity was expressed as 50% neutralization titer (NT50), which was determined using the half-maximal inhibitory concentration values of serum samples, normalized to control infections, from their serial dilutions. As shown in Fig. 2E, high homologous neutralizing antibodies levels were detected 3 weeks after priming the animals with one dose of 1 µg or 10 µg of GLB-COV2-043 mRNA-LNP vaccine, which was further increased by 3- (0.1 µg), 1.4- (1 µg) and 1.3- (10 µg) fold magnitude three weeks after the boost (week 6) sera. We further tested levels of heterologous neutralizing antibodies to understand the coverage of GLB-COV2-043 vaccine against the most current SARS-CoV-2 variants in circulation. Animals of all groups displayed mild to no neutralizing responses against SARS-CoV-2 Omicron BA.1 and XBB.1.5 expressing pseudoviruses on week 3, 3 weeks after one dose of vaccine, with mean NT_50_ values below the LLOQ (Fig. 2F,G). Post second immunization (week 6, 3 weeks post 2nd), groups receiving 1 µg and 10 µg doubled their levels of neutralizing antibodies against Omicron BA.1. The group receiving 0.1 µg of mRNA-LNP vaccine remained unresponsive (Fig. 2F). A very mild response of neutralizing antibodies against Omicron XBB.1.5 was detected in groups receiving the 2nd dose of 1 µg and 10 µg (Fig. 2G; week 6). These results indicate that GLB-COV2-043 mRNA-LNP vaccine was capable of inducing strong binding antibodies against SARS-CoV-2 spike from the Wuhan, Omicron BA.1 and XBB.1.5 strains. As expected, the homologous neutralizing antibodies titers were high. However, our GLB-COV2-043 mRNA-LNP vaccine, encoding Wuhan spike only, could not generate neutralizing antibody responses against Omicron BA.1 and XBB.1.5 variants.

### A booster dose of GLB-COV2-043 enhances antibody breadth, avidity, and virus neutralization capacity in mice

**Figure 3:**
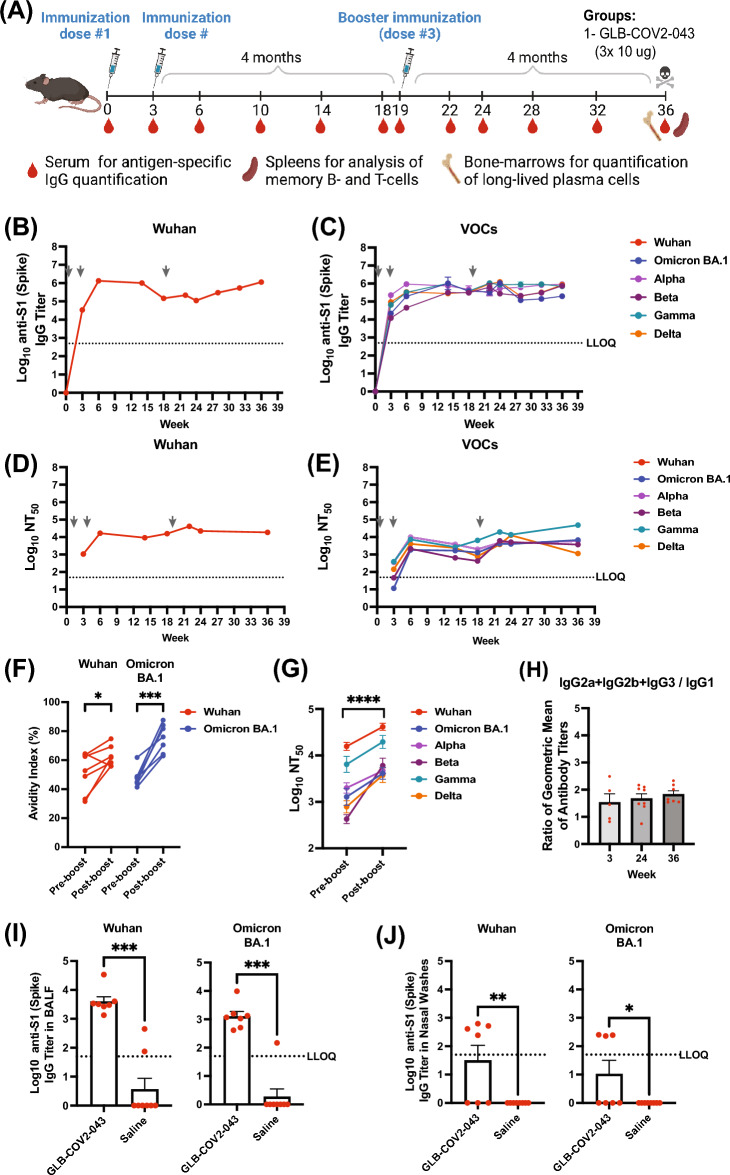
A booster dose of GLB-COV2-043 improves humoral immune responses by inducing antibody breadth, higher avidity, and virus neutralization capacity over time in mice. Mice were vaccinated intramuscularly with two 10 µg doses of GLB-COV2-043 mRNA-LNP, three weeks apart, followed by a booster dose 4 months later. (**A**) Scheme of immunizations and sample collection schedule. (**B**,**C**) End-point titers of antigen-specific serum IgG antibodies detected in sera collected from mice throughout study were evaluated by ELISA using soluble S1 recombinant spike proteins from (**B**) Wuhan, or (**C**) SARS-CoV-2 variants Alpha, Beta, Gamma, Delta and Omicron BA.1. (**D**,**E**) Anti-SARS-CoV-2 longitudinal neutralization antibody titers (NT_50_) evaluated using pseudovirus expressing spike protein from (**D**) Wuhan or (**E**) indicated variants. Grey arrows indicate immunization timepoints. (**F**) Levels of antigen-specific binding antibodies pre-boost (week 18) and post boost (week 36) were measured by ELISA, in presence or absence of chaotropic agent KSCN. The avidity index was expressed as the ratio between the area under the curve of the treated sample over non-treated sample. (**G**) Pairwise pre- and post-boost comparison of anti-SARS-CoV-2 neutralizing antibodies levels against Wuhan, Alpha, Beta, Gamma, Delta and Omicron BA.1 variants. (**H**) Sera collected at indicated timepoints was analyzed by ELISA for anti-SARS-CoV-2 S1 Spike-specific IgG1, IgG2a, IgG2c and IgG3, and titer ratios of IgG2a + IgG2b + IgG3 to IgG1 were calculated. (**I**,**J**) End-point titers of antigen-specific IgG antibodies detected in bronchoalveolar fluid (I) or nasal washes (**J**), collected from mice at the terminal timepoint of the study and evaluated by ELISA using soluble S1 recombinant spike proteins from Wuhan or Omicron BA.1 variants. Comparisons between pre- and post-boost were made by two-sided Wilcoxon signed rank test. *p < 0.05, ***p < 0.001, ****p < 0.0001.

Booster doses of the approved COVID-19 vaccines have been shown to improve protection against severe illness or death from infection. In addition, they improve neutralizing antibody responses against SARS-CoV-2 variants and vaccine effectiveness against COVID-19. Booster doses are safe and recommended for most people, including children^15,29^. In order to assess the effectiveness of a booster dose of GLB-COV2-043 mRNA-LNP vaccine in enhancing the immune response generated by primary vaccination, C57BL/6 mice were immunized with 2 intramuscular doses of GLB-COV2-043 (10 μg per animal per dose), 3 weeks apart, to reflect a primary vaccination schedule. Approximately 4 months after the second immunization (i.e., at week 19), animals received a booster dose of 10 μg of GLB-COV2-043 mRNA-LNP vaccine (Fig. 3A). Figure 3B,C shows homologous and heterologous binding antibody titers in sera derived from vaccinated mice, as measured by ELISA. Post-priming, on week 3, all animals immunized with GLB-COV2-043 mRNA-LNP vaccine presented high levels of serum IgG antibodies specific for the S1 domain of SARS-CoV-2 Spike protein from the Wuhan strain (Fig. 3B). As observed in the previous mice study, there was a significant increase in binding antibody titers following a second dose of GLB-COV2-043, measured at week 6 (3 weeks post second dose). Anti-spike serum IgG levels remained high, although a slight decline was observed at week 18. Following a booster immunization with 10 μg of GLB-COV2-043 at week 19, there was a gradual increase in serum antibody titers over time. Mice immunized with GLB-COV2-043 mRNA-LNP vaccine also developed robust binding antibodies to spike from all SARS-CoV-2 variants tested: Alpha, Beta, Gamma, Delta, and Omicron BA.1. Following a booster dose of GLB-COV2-043 at week 19, anti-spike serum IgG binding titers against all variants tested remained steadily high over > 4 months (Fig. 3C). Since, avidity is a semi-quantitative measure of the overall strength of the antigen–antibody complex, and is dependent on antibody affinity and valency, and antigen density^30^, we observed that the GLB-COV2-043 booster induced a marked increase in serum antibody avidity against both Wuhan and Omicron spike (S1) proteins (Fig. 3F). Our results are consistent with expected activation of germinal center B cells upon booster COVID-19 vaccination, which leads to increased antibody avidity over time.

The ability of anti-spike serum antibodies to neutralize in vitro infection by homologous (Wuhan) or heterologous (Alpha, Beta, Gamma, Delta, and Omicron BA.1) SARS-CoV-2 pseudotyped viruses was determined at selected time points (Fig. 3D,E). A marked increase in both homologous and heterologous neutralization titers was observed after animals received a second dose of GLB-COV2-043 during primary immunization series, measured at week 6 (3 weeks post second dose). Although homologous neutralization titers remained stable after primary immunization, heterologous titers against all variants tested steadily declined over time (Fig. 3E). Nevertheless, a booster dose of GLB-COV2-043 mRNA-LNP, given 4 months after the two-primary dose immunization, was able to significantly potentiate both homologous and heterologous serum neutralization titers against all variants tested (Fig. 3G).

One of the key aspects in protection against respiratory viruses is the generation of antibodies in mucosal surfaces. To investigate whether our vaccine induced any mucosal response, we evaluated binding antibody titers in bronchoalveolar lavage fluid (BALF) and nasal washes, collected from immunized animals at the terminal time point of the study (week 36, 4 months post boost). We observed that (intramuscular) immunization with GLB-COV2-043 induced significant levels of anti-SARS-CoV-2 IgG antibodies against both Wuhan and Omicron BA.1 spike antigens in BALF and nasal washes (Fig. 3I,J). Nevertheless, in line with what has been observed by others^31^, no mucosal IgA was induced by intramuscular immunization of our mRNA-LNP vaccine (data not shown).

### GLB-COV2-043 mRNA-LNP vaccine induces a T_H_1 biased immune response

**Figure 4:**
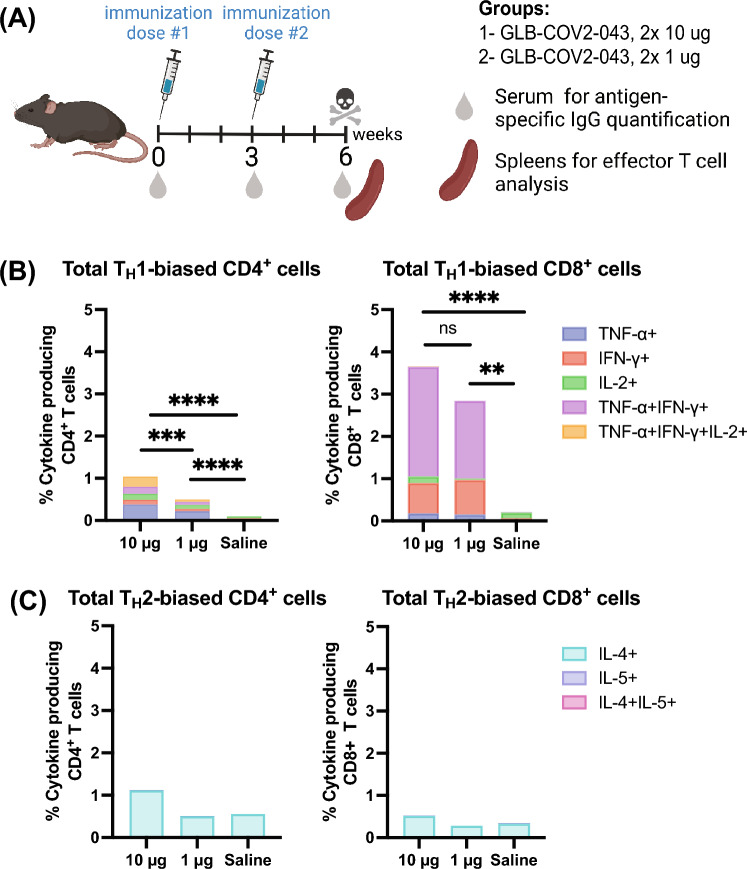
GLB-COV2-043 induces a T_H_1 biased immune response. C57BL/6 mice were immunized with 2 IM doses of GLB-COV2-043, at 1 μg or 10 μg per animal per dose, 3 weeks apart. (**A**) Scheme of immunizations and sample collection schedule. (**B**,**C**) Spleens were harvested 3 weeks after second immunization and stimulated with overlapping peptide pools from SARS-CoV-2 Spike protein, and percentage of T cells producing INF-γ, TNF-α, IL-2, IL-4 and IL-5 were measured by flow cytometry. Total poly-functional CD4^+^ and CD8^+^ T cells were studied for their (**B**) T_H_1- versus (**C**) T_H_2- biased responses based on their ability to secrete either INF-γ, TNF-α and IL-2 or IL-4 and IL-5. Comparisons between dosing groups were made by two-sided Mann–Whitney test. **p < 0.01 ***p < 0.001 ****p < 0.0001 ns: not significant.

T-cell responses correlate with protection against SARS-CoV-2 infection and are essential to define vaccine efficacy^32^. Current approved COVID-19 vaccines have been shown to elicit robust T-cell responses that may contribute to the control of infection and protect against severe disease, hospitalization, and death. A T_H_2/T_H_1 imbalance can lead to a multi-organ inflammatory response and is associated with higher COVID-19 mortality^33^. Therefore, in our studies, we examined the levels of pro-inflammatory and anti-inflammatory spike (S1) specific IgG subtypes, which are considered indicators of T_H_1 and T_H_2 responses. We measured the amounts of anti-inflammatory IgG1 and the pro-inflammatory IgG2a, IgG2b, and IgG3 anti-spike antibodies in the sera of mice immunized with 10 µg of GLB-COV2-043 mRNA-LNP, as per the dosing schedule outlined in Fig. 4A. We then calculated the ratio of geometric mean titers for the sum of IgG2a, IgG2b, and IgG3 relative to IgG1. We observed that the average ratio was consistently above 1 at all the time points analyzed (Fig. 3H), indicating a T_H_1-biased immune response.

To assess effector T cell responses, female C57BL/6 mice were divided into two groups, each receiving two intramuscular doses of LNP-formulated GLB-COV2-043 mRNA at either 10 μg or 1 μg, administered three weeks apart. 3 weeks after the second mRNA-LNP vaccine dose, spleens were collected, as depicted in Fig. 4A. Subsequently, spike-specific T CD4^+^ and T CD8^+^ responses were evaluated by measuring the intracellular production of T_H_1- and T_H_2-type cytokines through flow cytometry. Administering two doses of GLB-COV2-043 mRNA-LNPs, either at 10 μg or 1 μg, resulted in robust dose-dependent antigen-specific polyfunctional CD4^+^ and CD8^+^ T cell responses (as shown in Fig. 4B,C. Analysis of the intracellular cytokine profile in T cells showed that both 10 μg and 1 μg doses of GLB-COV2-043 mRNA-LNP vaccine induced strong T_H_1-biased, antigen-specific CD8^+^ and T CD4^+^ T cell responses. TNF-α and IFN-γ-producing CD8^+^ T cells were the most prominent, followed by CD8^+^ T cells that produced IFN-γ alone (as illustrated in Fig. 4B). We also assessed the profile of T_H_2 cytokines, IL-4, and IL-5, on CD4^+^ and T CD8^+^ T cells and found that GLB-COV2-043 mRNA-LNP induced minimal to baseline levels of T_H_2 T cells (Fig. 4C). T cells from control mice, immunized with saline, showed little to no cytokine production. Based on our analysis of IgG sub-classes and the T cell cytokine profile, we conclude that vaccination with LNP-encapsulated GLB-COV2-043 mRNA-LNP in mice results in a T_H_1-biased T cell response.

### GLB-COV2-043 induces antigen specific B (B_MEM_) and T (T_MEM_) cell memory in mice

**Figure 5:**
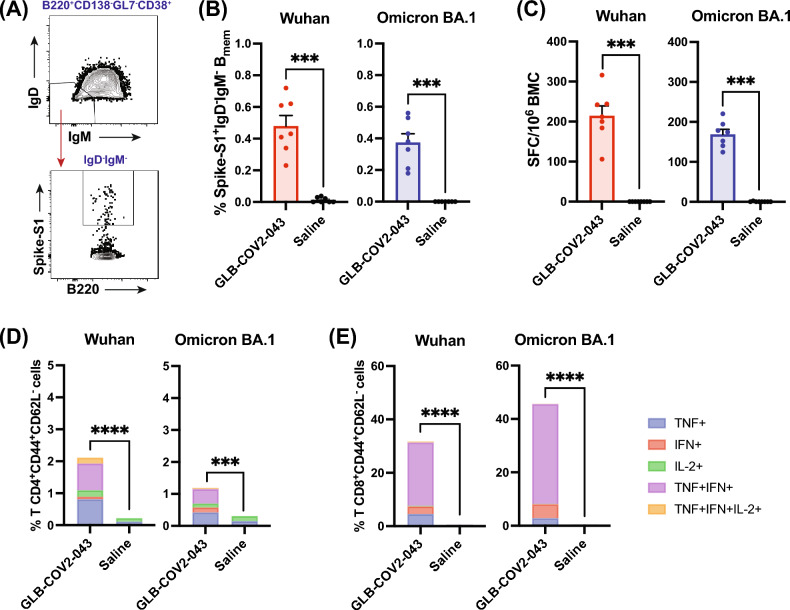
GLB-COV2-043 mRNA-LNP vaccine induces antigen specific B (B_MEM_) and T (T_MEM_) cell memory and long-lived plasma cells (LLPC) in mice. C57BL/6 mice were vaccinated intramuscularly with two 10 µg doses of mRNA-LNP GLB-COV2-043, 3 weeks apart, followed by a third dose 4 months later. Splenocytes were collected at week 36 (4 months post last dose) and evaluated by flow cytometry to detect spike-specific memory B and T cells. (**A**) Flow cytometry gating strategy for antigen specific B_MEM_ analysis. (**B**) Percentage of switched memory B cells, specific for S1 Spike antigen from Wuhan or Omicron BA.1 SARS-CoV-2. (**C**) Number of antigen-specific spot forming cells (SFC) from total bone marrow cells (BMC) of vaccinated animals, which are antibody (IgG) secreting long lived plasma cells, was quantified by ELISpot. (**D**,**E**) Percentage of effector memory (CD44^+^ CD62L^-^) CD4^+^ T (**D**) and CD8^+^ T (**E**) cells secreting either TNF-α, IFN-γ, IL-2, or any combination of these cytokines. Two-sided Mann–Whitney U-test. **p < 0.01, ***p < 0.001, ****p < 0.0001. Data is shown as Mean ± SEM.

Effective vaccines are expected to generate adaptive immunological memory in the B cell memory (B_MEM_) and T cell memory (T_MEM_) compartments^34–36^. This memory is crucial for quickly recalling immune responses upon re-exposure to the same virus or mutated variants, in order to prevent or resolve an infection and avoid severe outcomes for infected individuals^37^. The memory response of B cells relies on the differentiation of B_MEM_ into antibody secreting cells (ASCs) and Long-Lived Plasma Cells (LLPCs), while T cells are stimulated to produce cytokines that modulate the immune response. To evaluate if vaccination with 10 µg of GLB-COV2-043 mRNA-LNP could lead to long-term persistence of immune responses, we initially investigated the presence of spike-specific memory B cells and ASCs in circulation, and LLPCs in bone marrows, 4 months after completion of full vaccination schedule in mice. Presence of B_MEM_ cells specific for Wuhan and Omicron BA.1 spike (S1) antigens in splenocytes of vaccinated animals was detected by flow cytometry (Fig. 5A,B). No B_MEM_ cells were detected in animals vaccinated with placebo (saline). We further collected bone marrow cells from vaccinated animals and evaluated number of antibody (IgG) secreting cells by ELISpot. Vaccination with GLB-COV2-043 mRNA-LNP induced high levels of long-lived spike-specific bone marrow plasma cells, capable of recognizing both wild-type (Wuhan) and Omicron BA.1 spike antigens (Fig. 5C).

We next assessed whether GLB-COV2-043 mRNA-LNP vaccine candidate could elicit long term T cell memory. Splenocytes of animals vaccinated with either 10 µg of mRNA-LNP or placebo (saline) control were stimulated with overlapping peptide pools from the Wuhan or Omicron BA.1 SARS-CoV-2 full-length spike proteins, and subsequently analyzed by flow cytometry. High levels of effector memory (CD3^+^CD44^+^CD62L^-^) CD4^+^ and CD8^+^ T cells poly-functionally producing two or more T_H_1-type cytokines, TNF-α, INF-γ or IL-2, were readily detected in vaccinated animals (Fig. 5D,E). Conversely, levels of IL-4-secreting T_H_2 cells in vaccinated animals were comparable to what was observed in the placebo (saline) control group (data not shown). Collectively, these data show that GLB-COV2-043 mRNA-LNP vaccine candidate effectively induces long lasting antigen-specific memory B and T cell responses in mice.

### GLB-COV2-043 and a bivalent (Wuhan + Omicron BA.1) booster mRNA-LNP vaccine protect Hamsters against viral challenge and induce strong humoral immune responses

**Figure 6:**
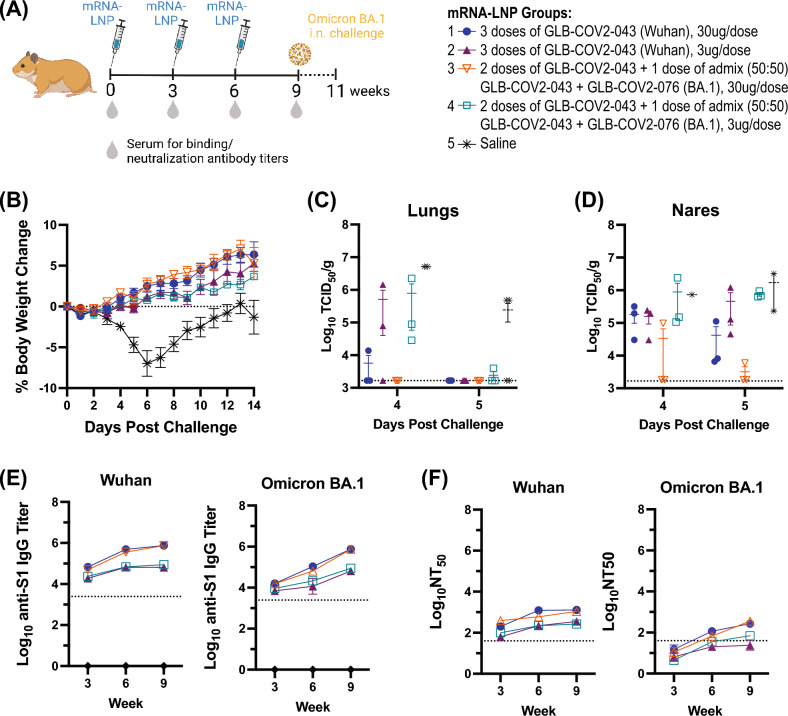
GLB-COV2-043 and GLB-COV2-076 mRNA vaccines protect Hamsters against challenge with Omicron B.1.1.529 (BA.1) live virus. Golden Syrian Hamsters were vaccinated intramuscularly with three doses of 30 µg or 3 µg of LNP-formulated mRNA vaccine, 3 weeks apart. Sera were collected for longitudinal serological evaluations on weeks 3, 6 and 9. At week 9, all Hamsters were intranasally challenged with SARS-CoV-2 Omicron BA.1 live virus and evaluated for viral load and clinical manifestations of COVID-19. (**A**) Scheme of dosing and sampling schedule. (**B**) Mean percentage change in body weight for each experimental group from the starting body weight measured on day 0 post challenge. (**C**,**D**) Infectious viral titers (TCID_50_) were measured in (**C**) lungs and (**D**) nares of challenged hamsters. (**E**) Longitudinal antigen-specific serum IgG antibody levels in vaccinated hamsters measured by ELISA against Wuhan and Omicron BA.1 S1 spike proteins. (**F**) Longitudinal serum neutralization antibody titers (NT_50_) evaluated using pseudovirus expressing spike protein from Wuhan or Omicron BA.1.

Evaluating and understanding the protective immune response against pathogenesis is crucial in vaccine development and preclinical evaluations. Syrian hamster model has proven to be useful for understanding SARS-CoV-2 pathogenesis and testing effectiveness of vaccines and anti-viral drugs^38,39^. Thus, we sought to evaluate the protective efficacy of GLB-COV2-043 prime followed by a bivalent booster of GLB-COV2-043 and GLB-COV2-076 mRNA-LNP against viral challenge in hamsters. GLB-COV2-076 mRNA encodes for Omicron BA.1 full-length spike antigen. Animals (n = 12) were immunized intramuscularly with 3 doses, given 3 weeks apart, of either 3 μg or 30 μg of the mRNA-LNP vaccine (Fig. 6A). Groups received either three doses of GLB-COV2-043 or two doses of GLB-COV2-043 followed by a single dose of bivalent (GLB-COV2-043 + GLB-COV2-076, 50:50 ‘bed-side’ mixed). 3 weeks post last immunization, all hamsters were challenged intranasally (IN) with SARS-CoV-2 Omicron BA.1 live virus and were followed for 14 days for viral load analyses and weight loss evaluations. All hamsters that received the mRNA-LNP vaccines were protected from weight loss (Fig. 6B). Conversely, hamsters in the placebo (saline) control group exhibited body weight reduction of approximately 8% on day 6 post-challenge, as expected in this animal model. Although they eventually regained weight, they did not recover to same weight of vaccinated groups (Fig. 6B). Furthermore, we measured viral load (TCID_50_/g) in lungs and nares of hamsters, infected with Omicron BA.1 virus 4–5 days post-infection. We observed that both monovalent GLB-COV2-043 and the bivalent booster GLB-COV2-043 + GLB-COV2-076 vaccines protected hamsters from viral replication in lungs and nares, when dosed at 30 μg per animal (Fig. 6C,D).

We subsequently measured longitudinal levels of anti-S1 Wuhan and Omicron BA.1 serum binding IgGs. As noted in Fig. 6E, all hamsters seroconverted after a single injection of GLB-COV2-043 vaccine, independent of the dose. Nevertheless, higher doses induced higher levels of antigen-specific IgG (Fig. 6E). Interestingly, the levels of anti-S1 SARS-CoV-2 Wuhan specific binding antibodies reached a plateau after the second injection of GLB-COV2-043 for all dose regimen, whereas the levels of anti-S1 SARS-CoV-2 Omicron BA.1 specific binding antibodies increased overtime (Fig. 6E). In addition, we assessed neutralizing antibodies titers through pseudovirus neutralization assays. We observed that, independent of the vaccine candidates used (monovalent GLB-COV2-043 or bivalent GLB-COV2-043 + GLB-COV2-076) and number of immunizations, all hamsters produced neutralizing antibodies against SARS-CoV-2 Wuhan spike (Fig. 6F). An increase in homologous neutralization titers was observed after the second dose, but thereafter titers reached a plateau and were not affected by the third booster immunization (Fig. 6F). The heterologous neutralizing antibody responses against Omicron BA.1 were lower, as previously described by others^40–42^. A single dose of GLB-COV2-043 mRNA-LNP vaccine did not induce heterologous neutralizing antibodies (Fig. 6F). However, hamsters that received a second 30 µg dose of GLB-COV2-043 showed detectable levels of neutralizing antibodies above the LLOQ. After the third booster dose, all hamsters in the groups that received the 30 µg dose, independent of the vaccine antigen, showed higher levels of neutralizing antibodies against Omicron BA.1. When combined, these data show that GreenLight Biosciences vaccine candidates, in prime-boost regimen, were efficacious against Omicron BA.1 viral challenge in hamsters and induced strong humoral immune responses against homologous and heterologous SARS-CoV-2 strains.

### GLB-COV2-043 mRNA-LNP vaccine candidate is safe in pre-clinical toxicity study in rats

**Figure 7:**
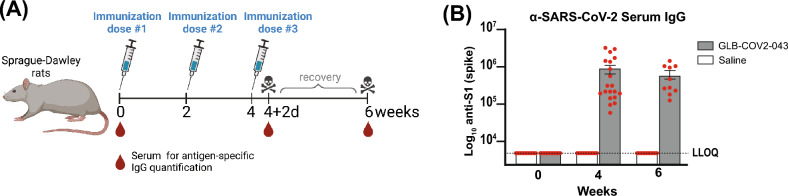
Levels of serum IgG titers against the S1 subunit of SARS-CoV-2 spike protein in immunized rats. Sprague Dawley rats were immunized with 3 doses of either GLB-COV2-043 (group 1) or Saline (group 2). (**A**) Vaccines were administered at 80 μg per animal every 2 weeks. (**B**) Serum samples were collected on week 0 (baseline), week 4 and week 6 for evaluation of anti-SARS-CoV-2 spike-S1 IgG levels by ELISA. Data is shown as Mean ± SEM.

To assess the pre-clinical safety of the GLB-COV2-043 mRNA-LNP vaccine candidate and the platform in general, we evaluated the toxicity of the formulated material in Sprague Dawley rats at the dose level of 80 μg, administered every 2 weeks for a total of three doses (Fig. 7A). All immunized animals seroconverted and generated high anti-S1 (Wuhan) serum IgG levels (Fig. 7B). The GLB-COV2-043 mRNA-LNP-associated non-adverse clinical observations included transient abnormal gait, decreased activity, reduced appetite and local irritation. Any reductions or gains in body weight were below 10% and correlated with reduction in food consumption for both male and female rats (data not shown). The non-adverse clinical pathology effects included transient changes in hematology, coagulation, clinical chemistry and alpha 2-macroglobulin levels (data not shown). These changes resolved during the recovery period, with the exception of alpha 2-macroglobulin, which was not considered adverse due to the magnitude, transient nature, and rate of recovery. The gross pathology findings that were present in the subcutis, administration site, and/or biceps femoris, correlated microscopically with moderate mixed cell inflammation. Overall, the effects attributed to GLB-COV-2-043 mRNA-LNP vaccine candidate are consistent with the immunological and inflammatory changes associated with the intramuscular administration of an immunogenic mRNA vaccine.

## Discussion

The multiple pre-clinical studies, described here, demonstrated the safety, immunogenicity, and protective efficacy of two mRNA vaccine candidates against COVID-19, developed by GreenLight Biosciences, in rats, mice and hamsters. Results show that both (GLB-COV2-043, as primary vaccine, and GLB-COV2-07,6 as part of GLB-COV2-043 + GLB-COV2-076 bivalent booster vaccine) vaccine candidates induced robust immune responses, protecting hamsters from SARS-CoV-2 Omicron BA.1 live virus challenge and COVID-19 disease severity. The studies in mice also showed that GLB-COV2-043 mRNA-LNP vaccine candidate induced strong and durable humoral immune responses that included binding anti-spike IgG antibodies and potent neutralizing antibodies activity against homologous and heterologous variants of SARS-CoV-2. These responses were durable and persisted for at least 4 months after a third booster vaccine dose. The durability of these immune responses were crucial for long-lasting protection and controlling infections^34^. These findings suggest that the GreenLight Biosciences mRNA-LNP vaccine candidates have similar pre-clinical immunogenicity and efficacy as the two approved mRNA vaccines, developed by Moderna and Pfizer-BioNTech^16,17,43^, which are currently being deployed globally to combat the COVID-19 pandemic.

T-cell responses are important to vaccine efficacy, and COVID-19 vaccines have been shown to induce robust cellular responses against SARS-CoV-2 in preclinical and clinical studies^5,16,17,44^. T helper (CD4^+^) cells are known to play an essential role by triggering the immune system to protect against SARS-CoV-2 via its production of cytokines^45^. They also play a critical role in protective immunity helping germinal center B cells produce antibodies against foreign pathogens^44^. We investigated the splenic total CD4^+^ and CD8^+^ T cell responses in vaccinated mice and showed that GLB-COV2-043 mRNA-LNP induces strong antigen-specific, polyfunctional, T_H_1-biased, acute and memory responses, evidenced by high levels of IFN-γ-, TNF-α-, and IL-2-secreting cells and only baseline levels of IL-4- and IL-5-secreting T cells. Importantly, a balanced T_H_1/T_H_2 immune response indicates that the vaccine candidate will have little to no potential of inducing vaccine-associated enhanced diseases (VAERD)^46,47^. We also observed that there is a T_H_1-biased IgG subtype response in vaccinated animals at all time points measured, which slightly increased over time and with boosting doses. This indicated that GLB-COV2-043 mRNA-LNP vaccine candidates elicited an IgG subclass trajectory consistent with a pro-inflammatory Fc response that is favorable for protection against SARS-CoV-2.

Vaccination with mRNA COVID-19 vaccines or exposure to SARS-CoV-2 have been shown to induce immunological memory in the B and T cell compartments^34,48,49^. Immunological memory responses provide long-term protection, allowing the immune system to recall a future re-encounter with the same pathogen or a mutated form of it. In COVID-19, immunological memory prevents disease severity and improves patients' prognosis and death^35,45^. We demonstrated that a third booster dose of GLB-COV2-043 mRNA-LNP vaccine induced strong splenic antigen-specific switched B_MEM_ cells against both Wuhan and Omicron BA.1 strains of SARS-CoV-2. This finding suggests that our vaccine candidate induced cells that may respond to a future encounter with the virus and its variants, which is supported by the presence of long-lived plasma cells in the bone marrow. Furthermore, we also observed generation of strong effector memory CD4^+^ and CD8^+^ T cell responses against both Wuhan and Omicron BA.1 strains of SARS-CoV-2. These T_MEM_ cells are poly-functional, evidenced by the production of up to three cytokines simultaneously, which has been shown to correlate with better vaccine protection^34,50–52^. The presence of B and T cell memory responses is indicative that the adaptive immune system of mice vaccinated with GLB-COV2-043 mRNA-LNP built capacity to not only recognize a SARS-CoV-2 and its variants but also to recall them, and it is indicative of the vaccine efficacy towards the protection of future related-SARS-CoV-2 infections.

In summary, we showed that the GreenLight Biosciences' GLB-COV2-043 mRNA-LNP vaccine candidate, encoding for the full-length SARS-CoV-2 Wuhan spike protein and an admix (50:50) of GLB-COV2-043 and GLB-COV2-076 [encoding for the Omicron (B.1.1.529) BA.1 full-length spike protein] mRNA-LNP vaccine candidates induced high antibody titers in mice and hamsters against multiple SARS-CoV-2 variants and provided robust protection against Omicron BA.1 live virus challenge in hamsters. In addition, GreenLight Bioscience's mRNA-LNP vaccine candidates induced long-term humoral and cellular immune responses. These data suggest that GLB-COV2-043 and GLB-COV2-076 mRNA-LNP vaccine candidates are immunogenic and protect against Omicron BA.1 live virus challenge. Finally, we demonstrated the pre-clinical toxicity of GLB-COV2-043 mRNA-LNP vaccine and found that they are safe and well-tolerated in SD rats, similar to other immunogenic mRNA COVID-19 vaccine. Through the demonstration of safety, immunogenicity and protective efficacy of our mRNA-LNP vaccine candidates against SARS-CoV-2, we establish that our modified mRNA and lipid-nanoparticle (LNP) delivery technology is a viable platform technology for future vaccine development against infectious disease targets, including emerging and re-emerging pathogens.

## Methods

### Ethics statements

All animal work related to this study was conducted following code of ethics for the care and use of animals as guided by the Public Health Service (PHS) Policy on Human Care and Use of Laboratory Animals, an in compliance with the Housing and handling of the animals following the standards of AAALAC (Association for Assessment and Accreditation of Laboratory Animal Care International). Studies were approved by institutional animal care and use committees (IACUC) at Charles River Laboratory and Bioqual Inc, ensuring that all experimental procedures were performed in compliance with applicable animal welfare laws and regulations. Animals were housed in suitable facilities with access to food, water, and environmental enrichment. Trained personnel performed the procedures, minimizing the number of animals used and optimizing their welfare. All mice were euthanized at terminal study timepoints by CO_2_ asphyxiation. Hamsters were euthanized via isoflurane inhalation and terminal bleed followed by bilateral thoracotomy. All methods were in accordance with ARRIVE guidelines.

### mRNA and LNP

GLB-COV2-043 mRNA encodes for the full-length wild-type spike (S) protein of SARS-CoV-2 from the original Wuhan strain, Wuhan-Hu-1 (GenBank: QHD43416.1). The mRNA is modified and contains pseudouridine (Ψ), instead of uracil (U). It is produced by in vitro transcription and purified to generate the final 2.0 mg/mL mRNA, which is stored at ≤ -65 °C until encapsulated in the lipid nanoparticle (LNP). The final formulated mRNA-LNP is manufactured in a multi-step process that involves mixing of aqueous GLB-COV2-043 mRNA and an organic phase of the lipid components (a proprietary cationic lipid, a saturated phospholipid, a PEG-lipid and cholesterol), of the lipid nanoparticles. After the LNPs are formed, the organic phase is removed, a buffer exchange is performed, sucrose is added, and the LNPs are diluted to the mRNA target concentration and aseptically filtered to generate the bulk mRNA-LNP dispersion. The mRNA-LNP is tested for critical attributes and stored at ≤ − 65 °C before final use. The lipid nanoparticle systems were developed by and licensed from Acuitas.

### Cell lines

HEK293FT (Thermo Fisher), were cultured in Dulbecco’s Modified Eagle’s Medium (DMEM, Gibco) supplemented with 10% FBS (Gibco), 1% penicillin–streptomycin (Gibco), 6 mM L-glutamine (Gibco), 1 mM MEM sodium pyruvate (Gibco), 0.1 mM MEM non-essential amino acids (Gibco) at 37 °C and 5% CO_2_. HEK293T-hACE2 cells (SBI Biosciences) were cultured in DMEM media containing 10% FBS, 1% PenStrep, and 2 µg/mL puromycin (Fisher Scientific) at 37 °C and 5% CO_2_.

### Cell surface expression and hACE2 binding assay

HEK293FT (Thermo Fisher) cells were transfected with GLB-COV2-043 mRNA encoding SARS-CoV-2 full-length wild-type (Wuhan-Hu-1) Spike protein using Lipofectamine MessengerMax (Thermo Fisher) following the supplier recommendations. For demonstration of cell surface expression of full-length Spike protein, 24 h post transfection, cells were collected and detected with anti-Spike rabbit IgG, which in turn were detected using anti-rabbit-IgG-FITC that showed surface expression of the full-length Spike proteins. For intracellular staining of the cells, DAPI was used. For hACE2 binding, 24 h post transfection, cells were collected and resuspended in FACS buffer. Cells were stained with biotinylated 1 × 10^–8^ M hACE2 (ACRO), in FACS buffer for 1 h at room temperature. Thereafter, cells were washed twice with FACS buffer and incubated with PE-Streptavidin (Invitrogen) in FACS buffer for 1 h at room temperature. The cells were washed and resuspended in FACS buffer, and the acquisition was performed on a BD Symphony instrument (BD Biosciences) and analyzed using FlowJo v10.8.1 software.

### Western blot

5 × 10^5^ HEK293FT cells/well were plated on a 6 well plate (Corning) and were transiently transfected with 2 µg GLB-COV2-043 mRNA encoding SARS-CoV-2 full length wild-type (Wuhan-Hu-1) Spike protein using Lipofectamine MessengerMax (Thermo Fisher) following the supplier recommendations. 20 h post transfection, the cells were lysed using RIPA lysis buffer (Thermo Fisher) with protease inhibitors (Thermo Fisher) and DNAse I (NEB). The cell lysates were collected after centrifugation (4 °C, 12,000 *g* for 15 min), reduced with DTT (Thermo Fisher) and denatured in sample buffer (Thermo Fisher) upon heating at 95 °C for 5 min. Cell extracts were run on a 4–12% Bis–Tris gel (Invitrogen) and transferred to a nitrocellulose membrane (Bio-Rad) using Trans-Blot Turbo transfer system (Bio-Rad). The membrane was then blocked with Intercept blocking buffer (Licor) for 1 h at room temperature and incubated with SARS-CoV-2 Spike RBD or anti-S2 antibodies (Sino Biological) at 4 °C overnight. The following day, the membrane was washed with 1 × PBS-0.2%Tween-20 (Fisher) and incubated with a HRP labeled goat anti-rabbit secondary antibody (Abcam) at room temperature for 1 h. The membrane was washed again with PBS-0.2% Tween-20 buffer, and the signal was detected using an Odyssey CLx Infrared Imaging System.

### Animal immunizations and viral challenge

C57BL/6 female mice, 6–8 weeks of age, were used for the studies that were performed at Charles River Laboratory (CRL). The protocol was conducted in compliance with CRL IACUC (Institutional Animal Care and Use Committee). Mice were separated into groups of 8 mice each (n = 8), bled on day-1 and immunized on day zero with three different intramuscular (IM) doses of 10 µg, 1 μg, or 0.1 µg of GLB-COV2-043 mRNA encapsulated in LNP. All mice received a second dose after 3 weeks. In one study, mice received a booster dose of 10 µg of mRNA-LNP, 4 months after primary immunization series. Monthly bleeds were performed to evaluate humoral immune responses over time, and the animals were euthanized 4 months after the booster dose, when tissues and spleens were collected for immune cells analysis. At terminal time points, blood and spleens were harvested for immune cell analysis.

Golden Syrian hamsters immunization and challenge studies were performed at BIOQUAL Inc. Handling samples and animals occurred in compliance with the biosafety protocols, and study was performed under an IACUC-approved protocol. 60 Golden Syrian hamsters were distributed into five groups of 12 animals each (6 female, 6 male). Animals were weighed and observed for clinical signs once daily during immunization days and before the live virus challenge. Hamsters received 3 IM doses, 3 weeks apart, of 30 µg or 3 µg of GLB-COV2-043 or an added (50:50) mix of GLB-COV2-043 (coding for wild-type Wuhan spike) plus GLB-COV2-076 (coding for Omicron BA.1 spike) mRNA-LNPs. Blood was collected for humoral immune responses analysis on each immunization day: 0, 21 (3 weeks), and 42 (6 weeks). On day 63 (9 weeks), animals were challenged via an intranasal route with 4.8 × 10^4^ TCID_50_ of SARS-CoV-2 Omicron B.1.1.529 (BA.1 variant). Post-challenge, clinical observations were recorded twice daily (AM/PM), and body weights were recorded once daily until termination. For longitudinal viral load analysis, oral swabs were collected on days 65, 67, 68, 70, and 76 or 77. On days 67, 68, 76, and 77. At euthanasia, all animals were necropsied for lung and nasal turbinate for viral load analysis and histopathology.

### Rat toxicity study

The Sprague Dawley (SD) rat repeat dose toxicity study was conducted at CRL. This cGLP study, based on approved protocol and led by an American Board of Toxicology certified toxicologist, complied with all applicable sections of the Final Rules of the Animal Welfare Act regulations (Code of Federal Regulations, Title 9), the Public Health Service Policy on Humane Care and Use of Laboratory Animals from the Office of Laboratory Animal Welfare, and the Guide for the Care and Use of Laboratory Animals from the National Research Council. A total of 30 rats, 15 males and 15 females, were used; 10 of those, from each sex, were used in the main study and 5 of those, from each sex, were used in the recovery phase of the study. The rats were intramuscularly immunized with 80 µg dose, administered once every two weeks for a total of 3 doses. Seroconversion was determined by quantitative ELISA assessment of anti-S (Wuhan) binding antibodies. The in-life procedures, observations (e.g., post-dose, food consumption, local irritation) and measurements (e.g., individual body weights) were performed for all main and recovery study animals. Clinical pathology included evaluation of samples for hematology parameters, coagulation parameters, clinical chemistry parameters and alpha-2-macroglobulin (α2M) analysis.

### Direct and avidity enzyme-linked immunoassay (ELISA)

ELISA 96-well plates (Thermo Scientific) were coated with 100 µL solution of the soluble recombinant Wuhan, Beta, Delta, Gamma, Alpha, or Omicron BA.1 Spike S1 subunit proteins (Sino Biological) in 1 × PBS (0.5 µg/mL) and incubated overnight at 4 °C. The wells were then aspirated and washed 3 times with 200 µL per well of 1 × PBS (Phosphate Buffered Saline). 200 µL of blocking solution (1% BSA/1 × PBS) were added to each of the wells and incubated for 2 h at room temperature. During the incubation period, mice serum samples were diluted with diluent solution (1% BSA, 0.05% Tween-20, 1 × PBS) starting at 1:500 and serially diluted 5-fold an additional 7 times to make a total of 8 dilutions. After the blocking incubation period, the blocking solution was discarded. The diluted mice serum sample solutions were added and incubated at room temperature for 2 h. Plates were washed 5 times with 200 µL of 0.05% Tween-20/1 × PBS. 100 µl of goat anti-mouse IgG H + L antibody conjugated to HRP (SouthernBiotech), diluted 1:5,000 in diluent solution, was added and incubated at room temperature for 1 h. Plates were washed 5 times with 200 µL of 0.05% Tween-20/1 × PBS. For detection development, 100 µl of TMB substrate (Fisher) was added to each well and the reaction was stopped after 15 min using 100 µl of 1 M H_3_PO_4_ (Sigma Aldrich). For avidity ELISA, one additional step was added in which half of the plates were incubated for exactly 12 min with 100 µl of 1.5 M KSCN (Sigma Aldrich) per well and the other corresponding plates were incubated with 1 × PBS under the same conditions. OD 450 readings were taken using the Biotek Synergy HTX plate reader and the data was analyzed using GraphPad Prism v.9.4.1. The end-point titers were calculated as the lowest dilution that emitted an optical density (OD) value greater than 4 times the background (secondary antibody alone).

### Pseudotyped SARS-CoV-2 neutralization assay

HEK293T-hACE2 cells (SBI Biosciences) were cultured in DMEM media containing 10% FBS, 1% PenStrep, and 2 µg/mL puromycin (Fisher Scientific) as a selection antibiotic at 37 °C and 5% CO_2_. Prior to plating cells, plates were coated with poly-d-lysine (Fisher Scientific CB-40210) at a concentration of 2.5 µg/well for 1 h at room temperature; poly-d-lysine was removed, and plates were washed with Gibco™ 1 × PBS (Thermo Fisher Scientific) and dried. Cells were plated with 1.25 × 10^4^ cells/well. The following day, fivefold serial dilutions of sera were made in HEK293T-hACE2 media with a starting dilution of 1:50 in a final volume of 50µL. 50µL of 1 × 10^7^/mL replication-deficient pseudovirus expressing spike of wild-type SARS-CoV-2 Wuhan or its variants Beta, Delta, Gamma, Alpha and Omicron B.1.1.529 (BA.1) and firefly luciferase within a Moloney Murine Leukemia Virus (MLV) backbone (eEnzyme) were added to each well and incubated together for 1 h at 37 °C and 5% CO_2_. Following incubation, the serum-pseudovirus mixture was added to the cells and incubated at 37 °C and 5% CO_2_ for 48 h. Each plate included wells with cell-only, as positive control, and pseudovirus-only, as negative control. Following incubation, media was removed and 50µL of Glo Lysis Buffer (Promega) was added to each well, incubated for 10 min, and transferred to an opaque white 96-well plate. 50 µL of Bright-Glo™ Luciferase Substrate (Promega) was added to each well and luminescence was determined using the GloMax® Navigator (Promega) with an integration time of 1 s. Relative luciferase units (RLU) were plotted and normalized in GraphPad Prism v.9.4.1 to the cell-only control as 100% neutralization and the pseudovirus-only control as 0% neutralization. A non-linear regression of log(inhibitor) versus normalized response with a HillSlope less than zero was used to determine the IC_50_ values.

### Enzyme-linked immunospot (ELISpot) assay

For the ELISpot assay, we followed the manufacturer protocol for mouse IgG B-cell ELISpot using an ELISpot Flex: Mouse IgG (ALP) kit (Mabtech). Briefly, sterile 96-well ELISpot plates with a PVDF membrane (Mabtech) were pre-treated with 15 μL of 70% ethanol and washed 5 times with 200 μL of sterile water per well. SARS-CoV-2 spike antigens (Sino Biological) were diluted to 5 μg/mL in sterile 1 × PBS. Coated plates were wrapped in parafilm to avoid evaporation and incubated overnight at 4 °C. The following day, plates were washed 5 times with 200 μL/well of sterile 1 × PBS to remove excess antigen and then blocked with 200 μL/well of R10 [RPMI 1640 (Fisher Scientific), 10% fetal bovine serum (FBS) (Fisher Scientific), 1% penicillin–streptomycin (Fisher Scientific)] for at least 30 min at room temperature. Cells were plated at 2.5 × 10^5^ cells/well in 100 μL using R10 and incubated in the coated plates for 16–24 h in a 37 °C humidified incubator with 5% CO_2_. The following day, the plates were washed 5 times with 200 μL/well of 1 × PBS. The detection antibody, anti-IgG-biotin, was diluted to 1 μg/mL in 1 × PBS-0.5% FBS and 100 μL was added to incubate for 2 h at room temperature. The 5 times wash step was repeated and 100 μL of streptavidin–alkaline phosphatase (ALP) diluted 1:1000 in 1 × PBS-0.5% FBS was added to each well. After incubating for 1 h at room temperature, plates were washed and 100 μL of 5-bromo-4-chloro-3-indolyl phosphate/nitro blue tetrazolium (BCIP/NBT) (Mabtech) was added to each well and incubated for about 5 min until spots emerged. The plates were washed with tap water extensively to stop color development and dried for 24 h. The spots were counted on an ImmunoSpot S6 Ultimate M2 analyzer (CTL), using ImmunoSpot 7.0.37.0 Professional DC program.

### Flow cytometry

Spleens were suspended in 1 × PBS and homogenized through a 70 μm strainer. Splenocytes were then incubated with ACK (Ammonium-Chloride-Potassium) lysis buffer and passed through a 40 μm strainer to prepare a cell suspension. Cells were plated at 5 × 10^5^ cells in 100 μl with T cell medium [RPMI 1640 (Fisher Scientific), 10% fetal bovine serum (FBS) (Fisher Scientific), 1% pyruvate (Fisher Scientific), 1% non-essential amino acids (NEAA) (Fisher Scientific), 0.4% MEM (minimum essential medium) vitamins (Fisher Scientific), 0.1% β-MercaptoethanoL (Fisher Scientific), dimethyl sulfoxide (DMSO, Sigma)]. This was followed by stimulating the cells with an overlapping peptide pool (JPT) diluted 1:200 in medium, or 1 × of a stimulation cocktail (eBioscience). After the cells have incubated at 37 °C, 5% CO_2_ for 2 h in the dark, media containing 1% of 10 × Brefeldin A (BFA) (Sigma) was added to each well. After 4 h incubation, FcR blocking antibody (Fisher Scientific), diluted to 1 µg/ml with 0.1% BFA, was added to the cells and incubated for 15 min at 4 °C in the dark. Cells were than stained for 30 min at 4 °C in the dark with viability and surface markers Zombie Aqua Fixable Viability Kit (Biolegend) or eFluor 780 Fixable Viability Dye (ThermoFisher), dump channel [anti- mouse CD19 (clone 6D5, Biolegend; clone 1D3, TONBO Biosciences), F4/80 (clone BM8, Biolegend; clone BM8.1, TONBO Biosciences), Gr-1 (clone RB6-8C5, Biolegend; clone RB6-8C5, TONBO Biosciences), CD11b (clone M1/70, TONBO Biosciences), CD11c (clone N418, TONBO Biosciences), anti-mouse CD3e (clone 145-2C11 Biolegend), CD62L (clone MEL-14, Biolegend), CD4 (clone RM4-5, Biolegend or TONBO Biosciences), CD44 (clone IM7, TONBO Biosciences), CD8a (clone 53–6.7, TONBO Biosciences). Subsequently, cells were fixed, permeabilized, then stained with intracellular markers anti-mouse TNF-alpha (clone MP6-XT22, Biolegend), IL-2 (clone JES6-5H4, TONBO Biosciences), IL-4 (clone 11B11, Biolegend), IL-5 (clone TRFK5, Biolegend), and IFN-gamma (clone XMG1.2, TONBO Biosciences). For antigen-specific B cell analysis, splenocytes were thawed using 37 °C pre-warmed R10 (10% FBS in RPMI 1640) and plated in R10 incubated overnight at 37 °C and 5% CO_2_. The following day, cells were plated at 5 × 10^6^ cells in R10 and stained for 30 min on ice in the dark with tetramers, composed of his-tagged spike protein (R&D Systems and BPS Biosciences) and labelled anti-His tag antibodies (clone J095G46, Biolegend). Cells were then stained for an additional 15 min with viability and surface markers Zombie Aqua Fixable Viability Kit (Biolegend), dump channel [anti- mouse CD4 (clone RM4-4, Biolegend), CD8a (clone QA17A07, Biolegend), Gr-1 (clone RB6-8C5, Biolegend), F4/80 (clone BM8, Biolegend), CD11b (clone M1/70, Biolegend), CD11c (clone N418, Biolegend)], anti- mouse GL7 (clone GL7, Biolegend), IgD (clone 11-26c.2a, Biolegend), B220 (clone RA3-6B2, Biolegend), CD138 (clone 281-2, Biolegend), CD38 (clone 90, Biolegend), CD19 (clone 6D5, Biolegend), IgM (clone 1B4B1, SouthernBiotech) and IgG1 (clone X56, BD Biosciences). The cells were analyzed using a BD FACSymphony A3 flow cytometer and data processed using FlowJo v10.8.1 software.

### TCID_50_ assay for SARS-CoV-2 infectious viral titers

Snap-frozen hamster tissue samples were homogenized in 2 mL ice-cold assay medium (DMEM + 10% FBS + Puromycin + P/S) for approximately 20 s using a hand-held tissue homogenizer (Omni International). The samples were centrifuged (300*g*, 4 °C, 10 min) to remove cellular debris. Clear flat-bottom 96-well culture microplates (BD Falcon) were seeded with Vero TMPRSS2 cells at 2.5 × 10^4^ cells per well in growth media (DMEM + 10% FBS + Puromycin + P/S) and incubated at 37 °C, 5% CO_2_ until 80–100% confluent. Growth media was aspirated out and replaced with 180 μL of diluent media (DMEM + 2% FBS + Puromycin + P/S) per well. Next, 20 μL of processed tissue sample was added to the top row of the plate in quadruplicate, mixed via pipetting, and then serially diluted down the rows by 20μL (tenfold dilution). Plates were incubated at 37 °C, 5% CO_2_ for 4 days. After incubation, the presence of cytopathic effects (CPE) in each well was recorded, and the TCID_50_ value was calculated using the Read-Muench formula.

### Statistical analysis

Arithmetic or geometric means are represented by symbols or the heights of bars, and error bars represent the corresponding SEM (Standard Error of the Mean). Dotted lines indicate assay's lower limits of quantification (LLOQ). Two-sided Mann–Whitney U-tests were used to compare two experimental groups and two-sided Wilcoxon signed-rank tests to compare the same animals at different time points. To compare more than two experimental groups, Kruskal– Wallis ANOVA with Dunn’s multiple comparisons tests were applied. For pseudovirus neutralization assay, a nonlinear regression of log(inhibitor) versus normalized response with a HillSlope less than zero was used to determine the IC_50_ values. Statistical analyses were done using GraphPad Prism v.9.4.1. *p < 0.05, **p < 0.01, ***p < 0.001, ****p < 0.000.

### Supplementary Information

Supplementary Figure 1.

## Data Availability

All data generated or analyzed during this study are already included in this published article. The raw data are not publicly available because they are proprietary to GreenLight Biosciences and Acuitas. GreenLight, through the corresponding authors, will make efforts to share additional information or details on reasonable request.
