# Supplementary material for: Safety, immunogenicity and efficacy of an mRNA-based COVID-19 vaccine, GLB-COV2-043, in preclinical animal models
Source: Sci Rep. 2023 Dec 1;13:21172. doi: 10.1038/s41598-023-46233-6 (PMC10692331; doi:10.1038/s41598-023-46233-6)
Supplement: Supplementary file 1 — Supplementary Figure 1. [file 41598_2023_46233_MOESM1_ESM.pdf]

Figure S1

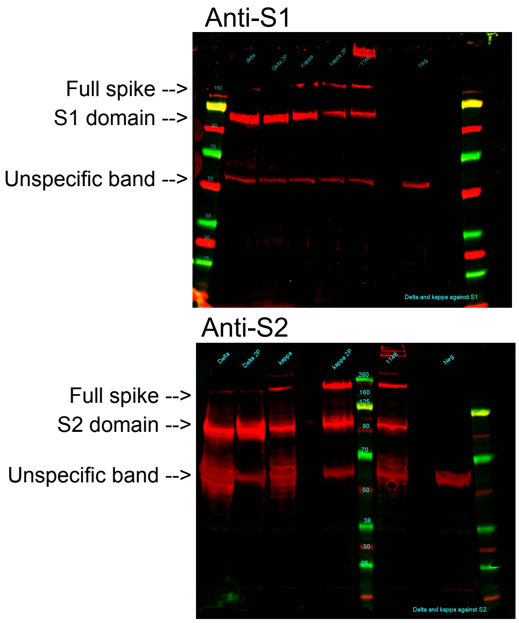

Figure S1: Raw images of Figure 1B. HEK293FT cells were transfected with mRNA expressing SARS-CoV-2 spike from indicated SARS-CoV-2 strains and evaluated by western blot 24 hours post-transfection using anti-RBD (upper panel) or anti-S2 domain antibodies (lower panel). Mock-transfected (PBS) cells served as a negative control. 2P = spike containing two stabilizing proline substitutions; 1148 = Wild-type (non-stabilized) SARS-CoV-2 Wuhan spike; Full spike, full length spike.
